# “Emerging Topics in Pain Medicine”: Advancing Research and Patient-Centered Health Strategies

**DOI:** 10.3390/jpm13081246

**Published:** 2023-08-10

**Authors:** Marco Cascella, Emiliano Petrucci, Franco Marinangeli, Alessandro Vittori

**Affiliations:** 1 Division of Anesthesia and Pain Medicine, Istituto Nazionale Tumori, IRCCS, “Fondazione G. Pascale”, 80131 Naples, Italy; 2 Department of Anesthesia and Intensive Care Unit, San Salvatore Academic Hospital of L’Aquila, 67100 L’Aquila, Italy; 3 Department of Anesthesiology, Intensive Care and Pain Treatment, University of L’Aquila, Piazzale Salvatore Tommasi 1, 67100 Coppito, Italy; 4 Department of Anesthesia and Critical Care, ARCO ROMA, Ospedale Pediatrico Bambino Gesù IRCCS, Piazza S. Onofrio 4, 00165 Rome, Italy

Pain, in all its various forms and manifestations, impacts the lives of millions worldwide [1], transcending age, gender [2], and cultural boundaries. It represents a significant concern, especially for the most fragile individuals. Nevertheless, the pursuit of suffering alleviation and to improving the quality of life for those affected by pain remains unwavering [1]. In this pursuit, we continuously seek to deepen our understandings of the complexities of pain, and identify innovative strategies to provide both relief and healing [3]. This process requires a multi-faceted approach that embraces research and healthcare strategies. For example, by investing in research, policymakers can promote evidence-based interventions that optimize pain relief and lead to personalized treatment approaches [4]. On the other hand, policy initiatives that prioritize pain management and interdisciplinary collaboration can foster an environment in which research findings translate into practical, patient-centered care [3].

Despite the evident gaps in pain management, the prospects for improvement are promising. As technological advancements and research progress, the field of pain medicine stands poised to offer a more comprehensive and individualized approach, in order to address the diverse challenges of pain and improve patients’ quality of life. For example, the aim of personalized approaches, i.e., catering to the unique needs of each patient, may encompass providing remote healthcare services via the utilization of telemedicine strategies. Within this framework, the field of pain medicine is witnessing notable expansion and advancement [5].

Education and advocacy both complement these efforts, empowering patients and healthcare professionals alike in the journey toward enhanced pain management [6,7]. Education in pain medicine plays a critical role in enhancing the understanding, assessment, and management of pain. Healthcare professionals may require specialized knowledge and skills to effectively address the diverse needs of individuals experiencing pain [8]. Moreover, in this field of medicine, advocacy is of paramount importance to raising awareness, promoting policy changes, and addressing disparities in pain management. Pain advocates work tirelessly to influence policymakers and legislators at various levels to recognize pain as a significant public health issue and improve access to quality care. They advocate for increased research funding to drive scientific advancements in pain management, and ensure the dissemination of evidence-based practices to healthcare providers and the public. Furthermore, patient support groups established and supported by these advocates can provide a valuable platform for individuals with pain to connect, find emotional support, and empower themselves while navigating their pain journey. Together, research, policy and education form a powerful alliance that seeks to provide relief and healing for all those living with pain [9].

The main obstacle in understanding and addressing pain lies in the fact that the term “pain” encapsulates an extensive and diverse range of clinical conditions [10]. Pain is not a singular, uniform experience, but rather a multifaceted phenomenon that can manifest in various forms and intensities across different individuals [1]. Ultimately, recognizing and embracing the diversity within the realm of pain is crucial for delivering patient-centered care and improving their quality of life. By acknowledging that pain is not a one-size-fits-all experience, healthcare providers can approach each patient with empathy, compassion, and a willingness to explore tailored solutions to address their unique pain challenges.

Effective pain management for specific categories, such as older adults and children, demands personalized and tailored approaches. When addressing pain in older adults, healthcare providers need to be highly attentive to age-related physiological changes, the presence of comorbidities, and the concurrent use of multiple therapies [11]. Therefore, striking a balance between pain relief and the potential risk of medication becomes critical in this population, in order to ensure optimal care [12]. On the other hand, pediatric pain management involves a multidisciplinary approach, enlisting the expertise of healthcare professionals from diverse disciplines [13,14,15]. This collaborative effort includes pediatricians, pediatric nurses, child life specialists, and psychologists, all working together to address the unique needs of young patients [16,17,18,19]. By combining their specialized skills, healthcare providers can offer comprehensive pain management strategies that consider not only the physical, but also the emotional and psychological well-being of the child [20], since children are not small adults, and newborns are not small children, but each of these patients has specific needs [21,22,23].

The heterogeneity of pain conditions is further amplified by the subjective nature of pain perception. Each individual’s experience of pain is influenced by their unique physiological and psychological makeup, as well as their emotional state and past experiences [24,25]. This subjectivity makes it challenging to develop a standardized approach to pain management that can effectively address the diverse needs of patients. A significant research avenue involves leveraging artificial intelligence (AI) techniques for automated pain assessment. This cutting-edge approach aims to enhance both the accuracy and efficiency of evaluating pain levels, offering promising prospects for improving pain management and patient care. By harnessing the power of AI, researchers seek to revolutionize pain assessment methodologies, ultimately leading to more personalized and effective interventions for individuals experiencing pain.

Cancer pain, existing on a continuum of severity and complexity [26], is an unwelcome companion for many individuals facing the challenges of cancer. From the early stages of diagnosis to the advanced phases of the disease, cancer pain can manifest in various forms, affecting patients physically, emotionally, and mentally [27]. This Special Issue tackles a variety of topics. The article authored by Becerra-Bolaños et al. [28] delves into the intricate relationship between pain and high-dose-rate brachytherapy in the context of treating locally advanced uterine cervical cancer. By assessing pain incidence and intensity, and examining patients’ satisfaction, the study offers valuable data to optimize pain management strategies during this critical phase of treatment. Their findings shed light on the importance of tailoring analgesic protocols, and pave the way for enhanced patient care and improved treatment outcomes.

Addressing the multifaceted nature of pain necessitates a collaborative effort across diverse medical disciplines. By amalgamating insights from different specialists, healthcare professionals can adopt a holistic approach to pain management, which encompasses pain relief, functional restoration, and an improved quality of life [29]. It is through the fusion of diverse perspectives, drawing from the wisdom of medical specialists, researchers, and the lived experiences of patients, that we can forge transformative breakthroughs in pain medicine [30]. In this complex scenario, promoting awareness and education on pain management is essential to empower patients and their caregivers to play an active role in their care journey [31,32,33]. Therefore, an enhanced understanding fosters open communication between patients and healthcare professionals, empowering patients to make informed decisions and actively participate in their pain management plan. On these bases, Cuomo et al. presented the results from a qualitative investigation on chronic low back pain (cLBP). This research delves into the concept of functional impairment in cLBP, which plays a pivotal role in determining patients’ quality of life and overall functionality. Remarkably, by gaining valuable insights both from the perspectives of different specialists involved in cLBP management and from the patients themselves, the study elucidates the complexities surrounding the assessment of functionality, and aims to bridge gaps in understanding, fostering more cohesive approaches to patient care. The strength of the study lies precisely in the evaluation of the patients’ perspectives.

In conclusion, “Emerging Topics in Pain Medicine” can serve as a critical cornerstone for advancing pain medicine research and embracing patient-centered health strategies. By recognizing the complex nature of pain and its profound impact on individuals, this Special Issue empowers researchers and readers to deepen their understanding of this intricate matter. Through ongoing efforts in research, policy advancements, education, and advocacy, we envision a future where pain management is personalized, compassionate, and accessible to all, ultimately bringing relief and healing to every individual living with pain.
